# Digital economy and carbon emission: The coupling effects of the economy in Qinghai region of China

**DOI:** 10.1016/j.heliyon.2024.e26451

**Published:** 2024-02-18

**Authors:** Tian Sun, Kaisheng Di, Qiumei Shi

**Affiliations:** a Department of EconomicsSejong University, Seoul 05006 South Korea; b Social Cooperation ServiceXi'an University of Finance and EconomicsXi'an 710100China; c College of Management and EconomicsTianjin UniversityTianjin 300072China; d College of Politics and Public AdministrationQinghai Minzu UniversityXining 810000China; e Department of Party CommitteeParty School of the Qinghai Provincial Committee of CPC Xining 810000China; f Health Education Services DepartmentXining Aier Eye HospitalXining 810000China

**Keywords:** Carbon dafeng, Digital economy, Regional economic development, Impact mechanism study

## Abstract

This study provides an in-depth analysis of the complex relationship between the digital economy and carbon emissions, fully drawing on essential principles of environmental economics, coupled economics, and sustainable development theory. Focusing on the Qinghai region in the western province of China, the study employs highly sophisticated methods such as multiple regression analysis and system dynamics modeling to reveal the multidimensional coupling effects between digital economy development and carbon emission dynamics. The study's results clearly show that in the Qinghai region of China, the booming growth of the digital economy is related to carbon emissions. Of particular interest, the study finds that this relationship exhibits a high degree of complexity and non-linearity and evolves gradually over time. Initially, the rapid expansion of the digital economy, accompanied by high energy consumption and increased carbon emissions, posed a significant challenge to environmental protection. However, a clear inverted “U"-shaped relationship has emerged as the digital economy evolves. This key inflection point signals a shift in the landscape as the digital economy begins to deliver some ecological benefits, potentially reducing the trend of carbon emissions in the future. The findings of this study go beyond simple causality and reveal a complex and evolving dynamic relationship between the digital economy and carbon emissions. Through such insights, this study provides a solid academic foundation and carefully constructs actionable policy recommendations to drive sustainable development. These insights apply to the Qinghai region of China and provide valuable references and lessons for other areas facing similar challenges.

## Highlights

• Examining the Complex Nexus between Digital Economy and Carbon Emissions.
• Leveraging Environmental Economics, Coupling Economics, and Sustainable Development Theory.
• Employing Multivariate Regression and System Dynamics Modeling for Thorough Analysis.
• Unveiling Multi-Faceted Coupling Effects, Including a Non-linear 'U'-shaped Relationship.
• Offering Insights and Policy Guidance for Sustainable Development in Qinghai and Beyond.

## Abbreviations full forms

DIUR: disposable income of urban residents
IGBR: urban-rural income gap
CPI: consumer price index
KPI: commodity price index
UUR: urban unemployment rate
TEDR: ratio of elderly to dependent youth
JPR: ratio of dependent adults
NOE: number of Internet users and Web

## 1 Introduction

The Environmental Kuznets Curve (EKC) theory, a significant concept in environmental economics, indicates that in the early stages of economic development, a positive relationship exists between income and environmental degradation, including the emission of pollutants and not just carbon emissions. However, this trend reverses beyond a specific income threshold [1]. Moreover, releasing carbon emissions represents a negative externality, imposing costs on society not shouldered by emitters themselves [2]. Interconnected economic systems underscore the intricate interconnectedness among diverse systems, emphasizing the cascading effects of changes within one system on others [3]. Importantly, high carbon emissions have been found to hinder economic progress, potentially hindering sustainable development trajectories [4]. While implicated in potential emissions escalation through their role in stimulating economic expansion, digitalization and technological advancement demonstrate the potential for mitigation. They facilitate carbon emission reduction through enhanced energy efficiency and optimized resource allocation strategies [5]. Sustainable development requires balancing economic growth, social equity, and environmental protection. Analysis suggests that integrating digitalization and technological advancement can facilitate the achievement of this balance by fostering the adoption of environmentally friendly practices in both production and consumption processes [6]. Balancing economic development and environmental conservation is crucial in effectively mitigating regional carbon emissions [7]. High levels of carbon emissions profoundly impact ecological systems and public health, posing a significant obstacle to achieving sustainable development goals. Conversely, implementing overly stringent measures on economic activities to curb carbon emissions may undermine economic development and social equity, demanding a delicate and nuanced approach to policy formulation and implementation [7].

Given the escalating global concern about climate change, policymakers and researchers are intensifying their focus on carbon emissions as a critical reference area [8]. The persistent increase in carbon emissions is expected to exacerbate the impacts of global climate change, potentially leading to severe repercussions for ecosystems and human health [9]. Due to its intricate nature, carbon emission reduction presents a complex challenge [10]. It is a multifaceted outcome resulting from the dynamic interplay among various factors, including but not limited to economic development, energy consumption patterns, technological advancements, and the formulation of relevant policies [11]. The complex and intertwined relationships among these factors create a highly intricate and dynamic system [12]. Understanding and managing carbon emissions necessitates a comprehensive evaluation that accounts for the multifaceted impact of diverse elements within the framework of this interconnected system [13]. Economic development, as a primary driver of carbon emissions, has garnered significant attention due to its profound implications for carbon emissions [14]. The precise role of digitization in influencing carbon emissions remains a subject warranting further exploration and analysis [15]. While digitization may indirectly contribute to increased carbon emissions through its role in stimulating economic growth, it also has the potential to mitigate emissions by fostering advancements in energy efficiency and facilitating optimized resource allocation [16]. Thus, a thorough and holistic examination of the role and impact of digitization is imperative for a comprehensive understanding and effective regulation of carbon emissions [17]. Notably, the implications of this interconnected system can vary across regions, particularly within China, which is recognized as the world's foremost carbon emitter. Significant disparities exist in economic development and carbon emissions among various areas within the country [18]. For instance, the dynamics of economic growth and carbon emissions in Qinghai, an inland province in China, may significantly differ from those observed in more developed and coastal regions [19]. Therefore, efforts to understand and manage carbon emissions must incorporate a comprehensive assessment of regional disparities and intricacies [20].

This paper conducts a comprehensive case study of the Qinghai region, aiming to examine the complex dynamics governing the influence of digitalization on carbon emissions and its interaction with economic development [21]. Analyzing panel data from 2005 to 2020 for Qinghai Province, a representative province in the western region, this study utilizes a dynamic spatial panel model and a mediating effects model. The primary objective is to investigate how the digital economy impacts emission reduction within the framework of economic coupling. The study aims to reveal the impact of economic agglomeration on emission reduction within the context of the digital economy, thereby establishing a solid theoretical basis. Examining the Qinghai region will provide new insights and a deeper comprehension of the intricate interplay among carbon emissions, economic development, and digitalization. Furthermore, it aims to illuminate the consequences of regional disparities within this interdependent system while offering a solid theoretical basis for developing effective strategies in carbon emission control. Through thoroughly exploring the interrelationship among carbon emissions, economic development, and digitalization in Qinghai, this study presents a new perspective and a systematic approach to understanding and managing carbon emissions.

The paper is structured into six main sections. The first section serves as an introduction to the research topic. The following section provides an extensive review of relevant literature. The third section explains the theoretical model and research hypotheses. Additionally, the fourth section outlines the research methodology and the precise procedures used for data collection. The fifth section explores the analysis and subsequent discussion of the findings. Finally, the sixth section presents the conclusions drawn from the study and suggests potential avenues for future research. The potential innovative contributions of this study can be briefly summarized as follows: the analytical focus from individual factors to their interconnected impacts, thus enhancing the scientific rigor of comprehensive factor analysis conducting empirical investigations into the simultaneous development of digital and regional economies, facilitating the achievement of carbon reduction objectives.3.Transitioning from an assessment of overall economic impacts to an examination of regional economic impacts, thereby enhancing the relevance and effectiveness of policy formulations.

## 2 Literature evaluation

### 2.1 Theoretical background

The evolution of the digital economy from the transformative impacts of information and communication technology propels societal development into the “network intelligence era" [21]. The rapid advancement of information technology has diversified and refined the digital economy, enabling the seamless integration of intelligence, knowledge, and creativity. This framework establishment underscores data's pivotal role as a critical production factor [22]. Moreover, the digital economy's ascendancy accentuates the economic globalization process, positioning it as the foundational force underpinning the third wave of globalization [23]. Recent global advocacy for environmental protection has significantly heightened concerns among businesses and residential communities regarding effective ecological management [24]. As the most populous developing nation, China faces an unparalleled challenge of effectively addressing climate change while ensuring the preservation of economic stability [25]. On one hand, the digital economy serves as a facilitator for the advancement of low-carbon development strategies. It is achieved by reducing transaction costs, mitigating resource disparities, and amplifying manufacturing efficiency [26]. Simultaneously, investments in digital technologies have the potential to stimulate technological progression and structural optimization, leading to the curtailment of resource and energy wastage. It fosters an environment conducive to green and sustainable productivity [27]. The growth of the digital economy encourages the regionalization of economic development. While the ubiquity of the internet bridges the gap commonly referred to as the “digital divide," it also inadvertently exacerbates the loss of fundamental rights through capital deprivation, catalyzing enterprises' decentralization [28]. The digital economy provides underdeveloped regions with accessible and affordable avenues for engaging with financial and economic services [29].

#### 2.1.1 Empirical studies

In recent decades, the escalation of environmental pollution and the challenges of economic transformation have gained increasing prominence in developing nations [30]. Notably, China grapples with a significant dependence on energy resources [31]. However, the emergence of the Industry 4.0 era has introduced the digital economy as a promising driver for sustainable development [32]. The application of digital technology in environmental preservation presents a fresh perspective for exploring avenues in green development [33]. Specifically, integrating information technology can establish a circular economy framework encompassing activities such as environmental monitoring, pollution control, information exchange, and waste re-utilization. This approach considerably curbs pollution emissions and minimizes energy consumption in traditional industries [34]. Concurrently, the digital economy spurs advancements in technology and the development of tools for environmental protection, including environmental engineering technologies, waste recycling technologies, and cleaner production methods [35]. Digital technologies transcend traditional geographical boundaries, optimizing the integration of resources [36]. The unrestricted flow of digital resources can foster a more profound division of labor and collaboration within regions, facilitating ecological cycles within ecosystems [37]. However, it is crucial to acknowledge that the excessive concentration of the digital economy may potentially exacerbate disparities in development and foster heightened competition within specific regions [38].

### 2.2 Gap in the literature

As global industrialization and modernization accelerate, annual human carbon emissions rise, particularly in developing and developed nations [39]. Numerous researchers consider carbon emissions a complex amalgamation of factors, encompassing not only energy consumption and industrial structure but also the extent of urbanization and population size [40]. Notably, the total carbon emissions within each region of China demonstrate a robust correlation with their respective energy consumption and levels of economic growth [41]. A substantial body of scholarly research has explored the intricate relationship between carbon emissions and economic development. Several modeling studies suggest that economic growth might, to some degree, catalyze an upsurge in carbon emissions [42]. However, the interplay between carbon emissions and economic development, particularly at the regional level, has yet to receive sufficient attention [43]. Recognizing this research gap, although some studies have examined the complex interplay among carbon emissions, economic development, and digitalization, they often overlook the intricate regional disparities, particularly in inland provinces such as Qinghai. Consequently, this paper undertakes a comprehensive study of carbon emissions within the Qinghai region, unveiling its intricate relationship with economic development and the influence of digitization within this context. A meticulous examination of these associations will yield a deeper understanding of the determinants of carbon emissions, thereby establishing a robust theoretical foundation for formulating effective strategies to mitigate carbon emissions.

## 3 Research hypotheses and theoretical mechanisms

The digital economy stands apart from the traditional economy under its reliance on information convergence, computing, and communication as primary catalysts [44]. It significantly aids in expanding e-commerce, developing novel competitive strategies, and adapting organizational structures and business operations to the digital landscape [45]. The digital economy can be characterized as a distinctive form where goods and services are made available at a digital discount [46]. The creation, distribution, and consumption of goods occur within a network of electronic channels known as the “digital economy," centered around advancements in digital technology and information networks [47]. The digital economy boasts a comparatively recent developmental trajectory and an evolving institutional framework [48]. The digital economy places more emphasis on the creation of novel goods and services rather than merely striving for increased productivity. Embracing a broad scope, the digital economy, as highlighted, encompasses multiple industries, diverse outputs (including goods and services), and various inputs (such as ICT technology-based production and distribution tools). Underscores that information serves as the primary resource within the digital economy, encompassing a plethora of economic and social activities conducted through the Internet and related technologies. Furthermore, it defines the term “digital economy" as a sector underpinned by digital technologies, including but not limited to e-commerce and e-business. The exploration of how the linking effects of the digital economy impact the global economic landscape and the continuous evolution of the digital economy itself has become a prevailing “hot topic" as the traditional economy transitions into the digital realm [49].

### 3.1 Digital economy development and regional economic development

Given the notably rapid expansion of the global digital economy, its emergence has increasingly positioned it as a significant and burgeoning economic force [50]. The roots of this emergence are multifaceted, stemming from political and economic factors and inherently grounded in the continuous progression of technological advancements shaped by broader sociocultural and global forces. The evolution of the Internet, serving as the bedrock for the digital economy's proliferation, played a pivotal role in the economic landscape of the 1990s. Nevertheless, the subsequent emergence of diverse information and communication technologies throughout the 2000s and 2010s spurred widespread economic transformation. These technological innovations have led to several pivotal digital changes, enabling individuals and organizations to utilize digital systems to assess potential actions within their operational context [51]. Notably, these transformations encompass virtualization, denoting the physical disentanglement of processes; reflecting the expansive growth of data storage capabilities; digitization, containing the conversion of all elements of the information value chain from analog to digital; and generativity, highlighting the application of data and technology in ways not initially intended, achieved through reprogramming and reorganization [52]. The impact of any technological advancement can be comprehended by gauging the extent of its diffusion and the breadth of its utilization [53]. As digital technology continues to increase and gain traction, even in emerging economies, its influence on the global economic landscape is rapidly gaining momentum [54].

**Fig. 1:**
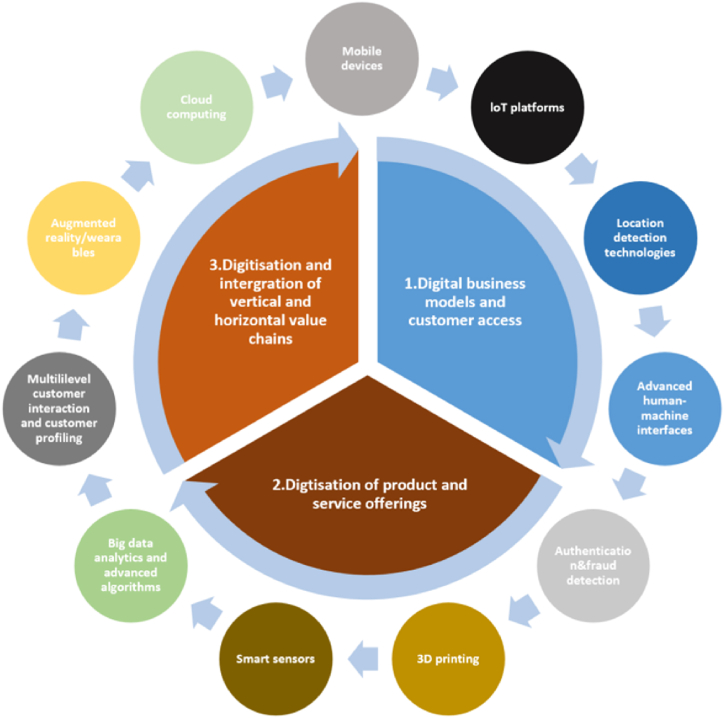
Industry 4.0 framework and contributing digital technologies.

The concept of the digital economy has materialized at the intersection of rhetoric and reality, establishing itself as a pivotal model. Recognized as a principal catalyst for economic expansion, it has engendered “far-reaching regional consequences on businesses, employment, and people," inducing transformative economic perturbations [55]. Particularly in developing nations, the digital economy holds substantial potential for invigorating economic progress, fostering heightened labor and capital productivity, reducing transactional expenses, and facilitating streamlined access to global markets [56]. These prospects are firmly grounded in empirical evidence, as the digital economies of emerging markets are consistently expanding at annual growth rates ranging between 15% and 25% [57]. The consequential impact can be delineated as a disruptive force, reshaping existing economic systems, processes, and sectors, thereby engendering shifts in consumer behavior, corporate relationships, and enterprise models [58]. This transformation can also be viewed as the emergence of novel economic systems, processes, and sectors. Although not achieving financial viability, emerging business models such as “Industry 4.0″ are progressively gaining prominence within contemporary discourses. Fig. 1 shows the Industry 4.0 framework and contributing digital technologies.

Technological tools like the Internet, big data, and artificial intelligence have significantly enhanced entrepreneurs' access to information, market intelligence, and resources, streamlining interaction and feedback with potential customers [59]. The proliferation of digital technologies has notably reduced innovation costs and diminished barriers to entrepreneurship, facilitating a surge in individuals launching their businesses through online platforms. This wave of innovation and entrepreneurial activity holds the potential to invigorate regional economies, expand employment prospects, and bolster regional competitiveness [60]. The evolution of the digital economy has revolutionized the functioning of conventional industries, prompting the optimization and modernization of industrial frameworks [61]. Leveraging digital technology enables traditional industries to achieve heightened production and management efficiency, improving overall output quality [62]. Such industries can adopt intelligent manufacturing practices, incorporate Internet of Things applications, and refine supply chain management through digital integration, ultimately enhancing their competitive edge. Simultaneously, the digital economy has given rise to new digital sectors such as e-commerce, internet finance, and the sharing economy, all of which have contributed positively to regional economic advancement [63]. For instance, e-commerce has introduced fresh prospects for the traditional retail sector, catalyzing its upgrade and transformation. The expansion of the digital economy has generated additional employment opportunities and has led to increased demands for skilled professionals, including data analysts, online marketing specialists, and artificial intelligence engineers [64]. Consequently, regions must intensify their efforts in talent training and recruitment, establish comprehensive talent development frameworks, and provide relevant educational and training opportunities to align with the evolving demands of the digital economy. Concurrently, the ascent of the digital economy also presents augmented prospects for self-employment, encouraging individuals to venture into entrepreneurship and innovation within this domain, thereby fostering an entrepreneurial culture and promoting regional development [65].

Hypothesis 1 (H1) The digital economy is driving the rapid development of the regional economy.

### 3.2 The relationship between digital economy development and carbon reduction

The digital economy's detrimental environmental externalities are a tangible concern [66]. Beyond reducing carbon emissions, the digital economy exhibits a “green blind spot" [67]. The proliferation of digital technology within the mining industry has expanded the scale of rare metals and mineral extraction, resulting in resource overconsumption and subsequent environmental degradation [68,69]. Moreover, the digital economy's development, spearheaded by major enterprises in telecommunications, software and information technology services, and the internet sector, consumes a substantial amount of power, with the sector accounting for 10% of global electricity consumption [70]. The industrialization of digital technology significantly contributes to power consumption, exacerbating the carbon emissions associated with China's heavy reliance on coal as its primary energy source [71,72]. As businesses strive to modernize their manufacturing facilities and bolster output through increased resource extraction and energy usage, carbon emissions are expected to rise [73]. However, the stability of business output during economic growth, coupled with the cost reduction in pollution management facilitated by digital technology, has the potential to mitigate CO2 emissions [74]. The current literature needs a comprehensive analysis of the mediating impact of the digital economy on low-carbon trade competitiveness and the interplay between digital technology advancement and green distribution development [75,76].Additionally, the role of information technology in facilitating China's energy sector to achieve carbon neutrality and the digital economy's contribution to transforming cities into low-carbon environments and evaluating policy implementation requires further exploration [77,78].

The rapid advancement of digital technologies presents a promising avenue for achieving a low-carbon economy. The energy, transport, and manufacturing sectors can be intelligently managed by leveraging digital and IoT technologies, reducing energy consumption and carbon emissions [79]. For instance, smart grids enable the intelligent distribution and management of energy, enhancing efficiency and minimizing energy waste [80]. Intelligent transportation systems optimize traffic flow, alleviate congestion, and decrease vehicle carbon emissions, while digital manufacturing facilitates refined production, waste reduction, and lower carbon emissions [81].Consequently, developing the digital economy provides technical support and pathways to expedite the realization of a low-carbon economy. The rise of the digital economy has facilitated energy transformation and the adoption of clean energy sources [82]. Digital technologies improve the predictability and controllability of renewable energy, leveraging data analysis and predictive models to enhance the accuracy and efficiency of wind and solar power generation [83]. Simultaneously, digital technologies facilitate distributed energy supply and intelligent energy management, including developing energy storage technologies and establishing smart grids [84]. Through digital and intelligent means, the energy transition progresses from traditional high-carbon energy sources to cleaner alternatives, effectively reducing carbon emissions. The development of the digital economy is intricately linked to carbon emission reduction [85]. The application of digital technologies supports the realization of low-carbon economic solutions, propelling the energy transition and encouraging the adoption of clean energy [86].The rise of the digital economy has contributed to the emergence of green consumption and the sharing economy, reducing resource waste and carbon emissions [87]. Digital technologies enable the monitoring and management of carbon emission data, facilitating the establishment of carbon markets and the implementation of carbon trading. The development of the digital economy also fosters the dissemination and application of sustainable development principles, promoting the implementation of sustainable practices [88].

Hypothesis 2 (H2) The development of the digital economy reduces carbon emissions and promotes sustainable economic development.

### 3.3 Digital economy and regional economic coupling concerning carbon reduction

The development of the digital economy plays a significant role in reducing carbon emissions by effectively optimizing resource allocation and enhancing resource utilization efficiency [89]. Through the application of digital technologies, there is an enhancement in the intelligence and precision of various processes, including production, transportation, and consumption, reducing both resource waste and energy consumption [90]. Implementing extensive data analysis, Internet of Things (IoT) technologies, and intelligent devices enables regional economies to gain better insights into energy consumption patterns and effectively manage energy supply and demand, consequently leading to a decrease in carbon emissions originating from energy sources [91]. An illustrative case is the establishment of smart grids and the utilization of innovative home technologies, which have enabled the intelligent monitoring and management of energy, resulting in improved energy efficiency and a subsequent reduction in carbon emissions [92]. Moreover, the advancement of the digital economy has stimulated innovation and the adoption of low-carbon technologies, providing a promising pathway for regional economies to achieve a reduction in carbon emissions. Notably, digital technology has facilitated the development and integrating of low-carbon technologies within various sectors, including energy, transportation, and manufacturing [93]. For instance, implementing intelligent transportation systems has effectively optimized traffic flow and reduced carbon emissions. At the same time, digital manufacturing techniques have enabled precise production and reduced waste generation and carbon emissions [94]. Additionally, the ascent of the digital economy has been instrumental in the emergence and adoption of clean energy and energy storage technologies, such as solar photovoltaic power, wind power, and electric vehicles, which have substantially contributed to reducing carbon emissions. These developments have significantly facilitated the transition of regional economies toward a low-carbon development trajectory [94].

The development of the digital economy presents a range of opportunities for establishing and advancing carbon markets, crucial for promoting effective measures in carbon emission reduction [95]. Digital technology plays a pivotal role in monitoring and managing carbon emission data, ensuring enhanced transparency and the effectiveness of emission reduction efforts. Leveraging digital platforms enables the efficient implementation of mechanisms for trading and pricing carbon emission rights, thus providing incentives for companies and institutions to reduce their carbon footprints actively [96]. Furthermore, the digital economy's progression has catalyzed innovative developments within the carbon market, including utilizing blockchain technology for secure transactions and the traceability of carbon emissions data [97]. Establishing robust carbon markets enables regional economies to effectively guide and incentivize enterprises, driving them to achieve their carbon reduction targets. In addition, the development of the digital economy has facilitated the dissemination and adoption of sustainable development concepts, thus fostering the transition of regional economies towards low-carbon development. Leveraging digital and internet technologies facilitates the broader dissemination of knowledge and information related to sustainable development, thereby increasing public awareness and participation in environmental protection and carbon emission reduction initiatives [18]. The rise of the digital economy has fueled the growth of green consumption and the sharing economy, effectively reducing resource waste and carbon emissions. With the support of digital technology, regional economies can achieve intelligent management and optimization of energy resources, promoting the use of renewable energy, enhancing overall resource efficiency, and reducing environmental pollution, all contributing to the realization of carbon emission reduction targets [17]. The development of the digital economy necessitates robust policy support, particularly in areas related to carbon emission reduction. Regional governments play a vital role in formulating and implementing policies that incentivize carbon emission reduction, such as introducing carbon pricing mechanisms and quota systems, to provide essential guidance and support for developing the digital economy. Governments can also reinforce the regulation of the digital economy, encouraging and guiding enterprises and organizations to adopt low-carbon technologies and business models and promoting the practical implementation of carbon emission reduction measures [98]. Additionally, the government can leverage digital technology to monitor and evaluate carbon emission reduction efforts, providing a solid scientific foundation for formulating and implementing effective policies.

Hypothesis 3 (H3) There is an inverted 'U' relationship between the coupling of the digital economy and the regional economy and carbon reduction.

## 4 Methodology

This research delves into an extensive comparative analysis of the expansion of the digital economy in Western provinces, with a particular focus on Qinghai Province. Situated in northwest China, Qinghai Province shares borders with Gansu, Xinjiang, Sichuan, and Tibet. Qinghai Province has emerged as a noteworthy example of a western Chinese province experiencing substantial growth in the digital economy. It stands out by surpassing the national average, with its digital economy contributing 8.1% to its GDP. Additionally, Qinghai Province boasts the distinction of being China's largest clean energy centre, responsible for 10% of the nation's total installed renewable energy capacity, with solar and wind power generation alone accounting for over 20%. A significant milestone was achieved in Qinghai Province in 2017, as it became the first Chinese province to attain carbon neutrality. This remarkable feat was accomplished by reducing two-thirds of the total carbon emissions and emission intensity. The pivotal role played by digital technology in Qinghai Province is evident in its facilitation of renewable energy production, utilization, and trade, leading to enhanced energy security, efficiency, and a reduction in the overall cost of carbon emissions. With its unique position as a trailblazer in the intersection of digital economy development and carbon neutrality, Qinghai Province is a critical case study for understanding the symbiotic relationship between digitalization and sustainable environmental practices.

The digital economy's current measurement by academic and governmental sectors is broadly categorized into three areas: value-added measurement studies, research on linked indexing, and the creation of satellite accounts. These categories serve as critical frameworks for understanding and evaluating the multifaceted dimensions of the digital economy. One prominent example of this approach is seen internationally with the Australian Bureau of Statistics (ABS) adopting the measurement method established by the U.S. Department of Commerce's Bureau of Economic Analysis (BEA). Through this method, the ABS has successfully quantified the value added by the Australian digital economy and its overall contribution to the national economy. Similarly, the BEA has meticulously defined the scope of the digital economy within the United States, conducting a comprehensive study utilizing the supply and use table to assess both the scale of the value-added and the total output of the U.S. digital economy. In China, the Chinese Academy of Social Sciences (CASS) and the China Academy of Information and Communication Technology (CAICT) have undertaken crucial initiatives to gauge the size of China's digital economy, emphasizing industrial digitization and digital industrialization. Various domestic and international agencies have adopted quantitative models and index assessment techniques to comprehensively measure data on relevant indicators associated with the functioning of the digital economy. Notably, globally recognized organizations such as the Organization for Economic Co-operation and Development (OECD), Eurostat, and the World Bank have established prominent statistical indices like the ICT and digital economy statistical index system, the Digital Economy and Society Index (DESI), and the Knowledge Economy Index (KEI), respectively. The World Bank's KEI research encompasses 146 nations, making it a significant global benchmark for evaluating the digital economy's progress and impact on national and international scales. By categorizing and utilizing these methodologies and indices, governments and research institutions worldwide can effectively monitor, evaluate, and strategize, developing and implementing policies conducive to the growth and sustainability of the digital economy.

**Fig. 2:**
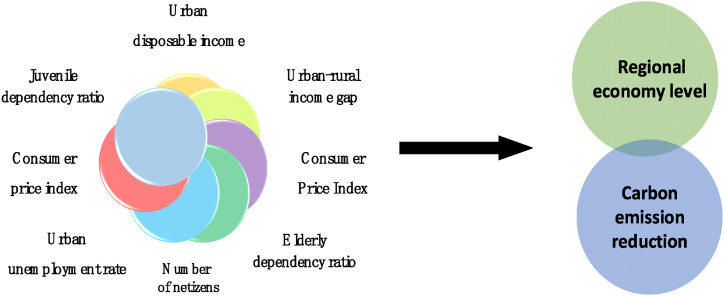
The theoretical model framework of the regional economy and carbon reduction driven by the digital economy.

Several exceptional and state-of-the-art measurement indicators have been developed in China to assess and analyze the digital economy's competitiveness. The Shanghai Academy of Social Sciences has created the Global Digital Economy Competitiveness Index, providing a comprehensive overview of China's position in the global digital economy landscape. The Caixin Think Tank has also introduced the China Digital Economy Index (CDEI), a crucial tool for understanding and evaluating the country's digital economy development. Tencent Research Institute's “Internet+" Index and Sadie Consulting's Digital Economy Index (DEDI) are also notable indicators offering nuanced insights into China's digital economy's multifaceted dimensions. Further research on creating satellite accounts has been a priority, with a particular focus on developing ICT and digital economy satellite accounts. This research has garnered significant attention from various international organizations, national government statistical agencies, and relevant academic institutions such as the Department of Economic and Social Affairs (DESA). The Australian Bureau of Statistics, Chilean Bureau of Statistics, Statistics South Africa, and Malaysian Bureau of Statistics have successfully established ICT satellite accounts, contributing to a comprehensive understanding of the digital economy within their respective contexts. The Organization for Economic Co-operation and Development (OECD) has actively addressed the complexities of measuring GDP within the digital economy, forming an advisory group and proposing the framework for the digital trade dimension and the basic framework for the digital economy satellite account. Experimental work has been conducted on compiling the supply and use table within the framework of DESA. Domestically, Chinese experts have conducted thorough studies on the essential characteristics of digital transactions within the satellite accounts, offering critical insights into the intricacies of China's digital economy landscape. The collaborative efforts of these organizations and institutions have significantly advanced the understanding and measurement of the digital economy within China and globally. Fig. 2 shows the theoretical model framework of the regional economy and carbon reduction driven by the digital economy.

### 4.1 Research approach

In this research, an autoregressive model employing time-series data is utilized, enabling an in-depth exploration of the dynamics of change and the impact of the digital economy on both the Qinghai region's economic growth and carbon emissions. The time series regression method differs from cross-sectional regression and is particularly effective in accounting for individual heterogeneity. This approach minimizes the covariance between dependent variables, ensuring a meticulous and comprehensive data analysis. The research employs multiple linear regression models to ascertain the linear relationship between the dependent variable and various independent variables. Weighted aggregation of the independent variables is employed to maximize the approximation's precision to the dependent variable's value. Subsequently, a likelihood ratio test is applied to assess the presence of heteroscedastic residuals. If the disturbances exhibit heteroskedasticity, alternative estimators prove more effective than the ordinary least squares (OLS) estimator, ensuring robustness in the analysis. Notably, careful attention is paid to endogeneity issues, identifying appropriate and reliable instruments. The selection of instruments hinges on their orthogonality to the errors and their correlation with the endogenous regressions. Robust evaluation of the fit of the first-stage regressions is pivotal in determining the instrumental variables' correlation with the included endogenous variables. Correspondingly, the moment conditions are critically assessed to ascertain the independence of the instrument from the unobservable error process. These methodological considerations lead to the results obtained through the multiple linear regression model, ensuring the rigour and accuracy of the empirical analysis.

$$Y=\beta_{0}+\beta_{1}X_{11}+\beta_{2}X_{21}+\ldots +\beta_{q}X_{q}+\varepsilon$$

The model can be written as a set of equations by separately substituting the sample with observed variables into it.

$$\left\{ \begin{array}{c} Y_{1}=\beta_{0}+\beta_{1}X_{11}+\beta_{2}X_{21}+\ldots +\beta_{q}X_{q1}+\varepsilon \\ Y_{2}=\beta_{0}+\beta_{1}X_{12}+\beta_{2}X_{22}+\ldots +\beta_{q}X_{q2}+\varepsilon \\ .\\ .\\ .\\ Y_{N}=\beta_{0}+\beta_{1}X_{1N}+\beta_{2}X_{2N}+\ldots +\beta_{q}X_{qN}+\varepsilon \end{array}\right.$$

This study develops a first-order autoregressive model based on multiple linear regression and the pertinent characteristics of the time series data.

$$Y_{t}=\beta_{0}+\beta_{1}y_{t-1}+\varepsilon_{i\;}\left(t=2,\ldots ,T\right)$$

Where the perturbation term is white noise that meets the conditions of homoskedasticity Var($\varepsilon_{i}$) = $\sigma_{\varepsilon }^{2}$, zero expectation E ($\varepsilon_{i}$) = 0, and zero autocorrelation Cov($\varepsilon_{t,}\varepsilon_{s}$) = 0,t≠s.

### 4.2 Data collection

The “digital economy" concept is multifaceted, with diverse assessment techniques and indicators utilized by different nations and institutions. Over 30 measurement tools and indicators have been developed internationally, including the OECD's digital economy statistics indicators, the EU's Digital Economy and Society Index (DESI), and China's ICT Digital Economy Accounting Methodology. This paper provides a comprehensive summary of the factors influencing the consumption behaviour of the digital economy, encompassing various metrics such as the per capita disposable income of urban residents (DIUR), the urban-rural income gap (IGBR), the consumer price index (CPI), the commodity price index (KPI), the urban unemployment rate (UUR), the ratio of elderly to dependent youth (TEDR), the ratio of dependent adults (JPR), and the number of Internet users and websites (NOE) in the region. The consumption level (Y1) and carbon emissions are critical metrics for assessing regional economic development (Y2). Given the complexity arising from the multitude of quantitative and qualitative categories involved in the factor analysis of the data, directly comparing the eight data indicators in terms of magnitude or quantitative level is only possible with the standardization of the data. Hence, data normalization was deemed necessary to mitigate the influence of varying magnitudes, with Table 1 presenting the characteristics of the processed data.

**Table 1:** Standardization results of evaluation indicators. Source: Calculated and summarized by the author.

Independent variable	N	Min	Max	AVG	SD	VS	SS	KS
DIUR	16.00	8271	35506	20406.25	8997.84	80961038.6	0.303	−1.234
IGBR	16.00	0.09	0.16	0.13	0.03	0.001	0.245	−1.126
CPI	16.00	100.80	110.10	103.54	2.41	5.812	1.582	2.519
KPI	16.00	100.40	110.60	102.88	2.61	6.811	2.005	4.433
UUR	16.00	2.14	3.92	3.15	0.61	0.377	−0.233	−1.36
TEDR	16.00	27.16	36.12	30.94	2.66	7.05	0.619	−0.467
JPR	16.00	8.47	12.10	9.84	1.01	1.016	1.156	1.134
NOE	16.00	12	207	83.38	68.37	4674.65	0.69	−1.068

Before constructing the structural equation model, exploratory factor analysis was conducted on the data scale to assess the data's validity. This step was undertaken to ensure that the data from the institutional aspect of the study was grounded in scientific principles and aligned with theoretical assumptions. The outcomes of the validation process, presented in Table 2 using SPSS (27) results, indicated the suitability of the dimensions for the reliability study. The high Kaiser-Meyer-Olkin (KMO) value of 0.734, Bartlett's sphericity test chi-square value of 186.529, and a significant level of 0.001 underscored the significance of the data quality. Furthermore, the factor loading matrix and variance contribution ratio tests demonstrated strong structural validity, with values consistent with and surpassing the predefined threshold, thereby affirming the logical coherence of the model's surface data from a scientific perspective.

**Table 2:** Evaluation index test results. Source: Calculated and summarized by the author.

KMO laboratory value		0.734
Bartlett sphericity test	Approximate Chi-square	186.529
	df	28.000
	P-value	***

***p < 0.001.

This study uses factor analysis to extract the factors, as indicated in Table 3. The factor load matrix computation is the essential component of the factor analysis method, and its calculation results vary depending on the calculation method.

**Table 3:** Common factor variance of evaluation index. Source: Calculated and summarized by the author.

Standardized variate	Initial value	Extraction value
Z (DIUR)	1	0.959
Z (IGBR)	1	0.781
Z (CPI)	1	0.994
Z (KPI)	1	0.966
Z (UUR)	1	0.905
Z (TEDR)	1	0.584
Z (JPR)	1	0.768
Z (NOE)	1	0.976

Extraction method: Principal component analysis.

The common factor variance describes the data information derived from the variables; typically, a value greater than 0. 5 implies that it can be stated. The juvenile dependency ratio is the only variable in Table 3 whose extracted values are less than or equal to 0.7. The cumulative variance contribution is displayed in Table 4; Component 1's factor has a variance contribution of 66.601%, while Component 2's has a variance contribution of 20.056%. These two factors together have a cumulative variance contribution of 86.657%, so two common factors are chosen to achieve the effect of dimensionality reduction.

**Table 4:** Cumulative variance contribution rate. Source: Calculated and summarized by the author.

Ingredient	Initial eigenvalue			Extract the sum of load squares.		
	Total	Percentage of variance (%)	Cumulative percentage (%)	Total	Percentage of variance (%)	Cumulative percentage (%)
1	5.328	66.601	66.601	5.328	66.601	66.601
2	1.604	20.056	86.657	1.604	20.056	86.657
3	0.870	10.870	97.528			
4	0.088	1.098	98.626			
5	0.055	0.693	99.319			
6	0.038	0.471	99.79			
7	0.011	0.132	99.922			
8	0.006	0.078	100			

Extraction method: Principal component analysis.

**Fig. 3:**
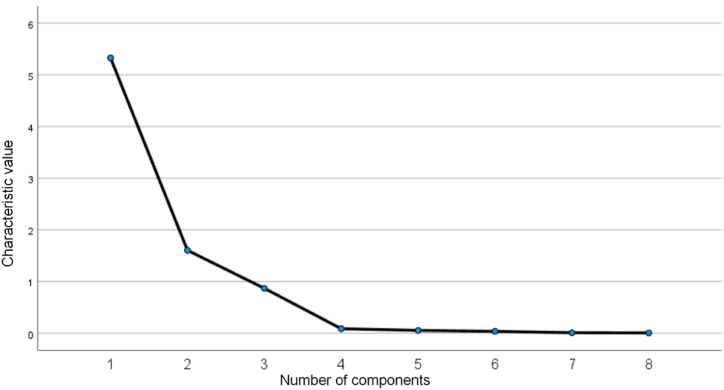
Factor analysis debris diagram. Source: Calculated and summarized by the author.

The first and second factors' connection lines fall steeply. In contrast, the other factor connection folds exhibit a flatter trend, as seen from the factor analysis rubble plot in Fig. 3. It illustrates the ability of the first and second factors to indicate the overall variance.

As shown in Table 5, the per capita disposable income of urban residents (DIUR) factor is 0. 959, the old age dependency ratio (TEDR) factor is 0. 630, and the juvenile dependency ratio (JPR) factor is 0. 873. The number of internet users and websites (NOE) factor is 0. 962, which has a significant loading. Table 5 displays the rotated component matrix. Since the consumer price index (CPI) and commodity price index (KPI) are 0. The second factor is attributed to price, 981 and 0. 968, respectively. As a result, the first factor is attributed to disposable income and the e-commerce environment. The findings from the research and analysis are used to describe the state of digital consumption in China's digital economy.

**Table 5:** **Rotating component matrix. Source: Calculated and summarized by the autho**r.

Independent variable	Component 1	Component 2
DIUR	0.959	−0.200
IGBR	−0.860	0.204
CPI	−0.177	0.981
KPI	−0.170	0.968
UUR	−0.937	0.164
TEDR	0.630	−0.433
JPR	0.873	−0.071
NOE	0.962	−0.225

Extraction method: Principal component analysis.

## 5 Analysis and discussion

### 5.1 Analysis

The criteria may be established when the processes mentioned above have been taken. The factor scores corresponding to the number of economic variables can be determined using the matrix of component score coefficients. The factor score function is created by substituting the initial variables for the linear equation representation of the principal component factors. The factor scores corresponding to the number of economic variables can be determined using the matrix of component score coefficients. As indicated in Table 6, the factor score function is created by substituting the original variables for the principal component factors' linear equation representation.

**Table 6:** Component score coefficient. Source: Calculated and summarized by the author.

Independent variable	Component 1	Component 2
DIUR	0.219	0.049
IGBR	−0.193	−0.03
CPI	0.115	0.508
KPI	0.115	0.502
UUR	−0.220	−0.065
TEDR	0.094	−0.133
JPR	0.218	0.105
NOE	0.216	0.036

Extraction method: Principal component analysis.

From Table 6, the linear relationship between the 2 common factors and each indicator can be derived as follows.

$$F1=0.219Z_{\left(\text{DIUR}\right)}-0.193Z_{\left(\text{IGBR}\right)\;}+0.115Z_{\left(\text{CPI}\right)}+0.115Z_{\left(\text{KPI}\right)}\;-0.220Z_{\left(\text{UUR}\right)}+0.094Z_{\left(\text{TEDR}\right)\;}+0.218Z_{\left(\text{JPR}\right)}+0.216Z_{\left(\text{NOE}\right)}$$

$$F2=0.049Z_{\left(\;\text{DIUR}\right)}-0.\;03Z_{\left(\text{IGBR}\right)}+0.508Z_{\left(\text{CPI}\right)}+0.\;502Z_{\left(\text{KPI}\right)}-0.\;065Z_{\left(\text{UUR}\right)}-0.133Z_{\left(\text{TEDR}\right)}+0.105Z_{\left(\text{JPR}\right)}+0.036Z_{\left(\text{NOE}\right)}$$

A statistical technique for examining the interdependence of variable interactions is regression analysis. Explaining the variance across variables illustrates how two or more explanatory factors (1st principal component score, second principal component score) impact the explained variables. After regressing the explanatory variables (Y1) and the explanatory variables (1st principal component score, second principal component score), the principal component scores are created by multiplying the corresponding factor scores by the arithmetic square root of the corresponding variance. The results are displayed in Table 7.

**Table 7:** Results of regression analysis. Source: Calculated and summarized by the author.

Model	Standard coefficient	Non-standard coefficient		t	P-value
		Standard error	B		
(Constant)	–	494.851	11372.937	22.983	***
F1	0.910	511.080	5041.055	9.864	***
F2	0.246	511.080	1361.676	2.664	**

***p < 0.001, **p < 0.01.

As a result, the following outcomes are possible: Z(Y) = 0. 910F1+0. 246F2. The principal component regression coefficient vectors and the matrix made up of the coefficient vectors of the first two components are estimated to produce the following matrix:

$$\left(\begin{array}{c} \;0.219\\ -0.193\\ \;0.115\\ \;0.115\\ -0.220\\ 0.094\\ \;0.218\\ 0.216 \end{array}\begin{array}{c} \begin{array}{c} \;0.049\;-0.030\;\\ \;0.508\\ \;0.502\\ -0.065\; \end{array}\\ -0.133\\ \;0.105\\ \;0.036 \end{array}\right)\left(\begin{array}{c} 0.910\\ 0.246 \end{array}\right)=\left(\begin{array}{c} \begin{array}{c} \begin{array}{c} \begin{array}{c} \;0.211\;-0.183\\ \;0.223 \end{array}\\ \;0.228 \end{array}-0.216\\ \;0.053\\ \;0.224 \end{array}\\ \;0.205 \end{array}\;\right)$$

Based on the above results, the regression equation for the independent variables was derived:

$$Z_{\left(Y1\right)}=0.211Z_{\left(\text{DIUR}\right)}-0.183Z_{\left(\text{IGBR}\right)}+0.223Z_{\left(\text{CPI}\right)}+0.228Z_{\left(\text{KPI}\right)}-0.\;216Z_{\left(\text{UUR}\right)}+0.053Z_{\left(\text{TEDR}\right)}+0.224Z_{\left(\text{JPR}\right)}+0.205Z_{\left(\text{NOE}\right)}$$

According to the regression results, all independent factors positively impacted residents' consumption levels, except for the urban-rural income disparity and unemployment rate. Consumer consumption level in Qinghai will rise by 0.221%, 0.223%, 0.228%, 0.053%, 0.224%, and 0.205% for every 1% increase in the disposable income of urban residents (DIUR), consumer price index (CPI), commodity price index (KPI), the old-age dependency ratio (TEDR), and several internet users (NOE) variables. The significance of digital services is demonstrated by the number of Internet users positively impacting spending on digital consumption. By connecting networks, logistics, and commodities, the digital development of regional economic development realizes the efficient allocation of regional economic resources, thereby giving regional economic development—especially in Qinghai, an ecologically conscious province—a significant leading and demonstration role. Now, we will examine the connection between carbon emissions and the digital economy. It illustrates the variance across variables by illustrating the level of influence of two or more explanatory factors on the explanatory variable (1st principal component score, second principal component score). Then, the explanatory variables (Y2) and the explanatory variables (1st principal component score, second principal component score) are regressed, and the results are displayed in Table 8. The principal component scores are obtained by multiplying the corresponding factor scores by the arithmetic square root of the corresponding variance.

**Table 8:** Results of regression analysis. Source: Calculated and summarized by the author.

Model	Standard coefficient	Non-standard coefficient		t	P-value
		Standard error	B		
(Constant)	–	48.082	2723.481	56.643	***
F1	−0.935	49.659	−762.092	−15.347	***
F2	−0.280	49.659	−228.435	−4.600	***

***p < 0.001.

As a result, the following outcome is possible: Z (Y2) = −0. 935F1-0. 280F2. The outcome matrix was created by estimating the matrix made up of the coefficient vectors of the first two components and the vectors of the principal component regression coefficients:

$$\left(\begin{array}{c} \;0.219\\ -0.193\\ \;0.115\\ \;0.115\\ -0.220\\ 0.094\\ \;0.218\\ 0.216 \end{array}\begin{array}{c} \begin{array}{c} \;0.049\;-0.030\;\\ \;0.508\\ \;0.502\\ -0.065\; \end{array}\\ -0.133\\ \;0.105\\ \;0.036 \end{array}\right)\left(\begin{array}{c} -0.935\\ -0.280 \end{array}\right)=\left(\begin{array}{c} \begin{array}{c} \begin{array}{c} \;-0.218\;\\ \;0.188 \end{array}-0.250-0.248\\ \;0.187 \end{array}-0.051-0.233\\ \;-0.212\; \end{array}\;\right)$$

The following results were used to create the regression equation for the independent variables:

$$Z_{\left(Y2\right)}=-0.218Z_{\left(\text{DIUR}\right)}+0.188Z_{\left(\text{IGBR}\right)}-0.250Z_{\left(\text{CPI}\right)}-0.248Z_{\left(\text{KPI}\right)}+0.187Z_{\left(\text{UUR}\right)}-0.051Z_{\left(\text{TEDR}\right)}-0.233Z_{\left(\text{JPR}\right)}\;-0.212Z_{\left(\text{NOE}\right)}$$

The regression analysis indicates that all factors, except for urban residents' disposable income (DIUR) and the urban unemployment rate (UUR), harm carbon emission levels. In Qinghai, an increase of 1% in the income gap between urban and rural areas (IGBR), disposable income of urban residents (DIUR), consumer price index (CPI), commodity price index (KPI), the old-age dependency ratio (TEDR), the juvenile dependency ratio (JPR), and the number of Internet users (NOE) results in a reduction of 0.218%, 0.250%, 0.248%, 0.051%, 0.233%, and 0.212% in carbon emissions, respectively. The analysis highlights the pivotal role of digital economy growth in guiding regional carbon emission reduction, aligning with critical policy objectives for the development of the Western region. This significance underscores the Western region's leadership in achieving carbon emission peaking. A comprehensive examination of the indicator system's design and operational framework confirms the validity of the proposed hypotheses. The current assessment of Qinghai's local situation is a representative example of the trends observed in other western provinces. Presently, Qinghai has transitioned beyond the phase where the digital economy contributed to increased CO2 emissions, promoting the adoption of carbon emission reduction technologies and fostering local and regional economic growth, marking the progression through the first stage of the inverted U-shaped relationship.

### 5.2 Robustness tests

**Fig. 4:**
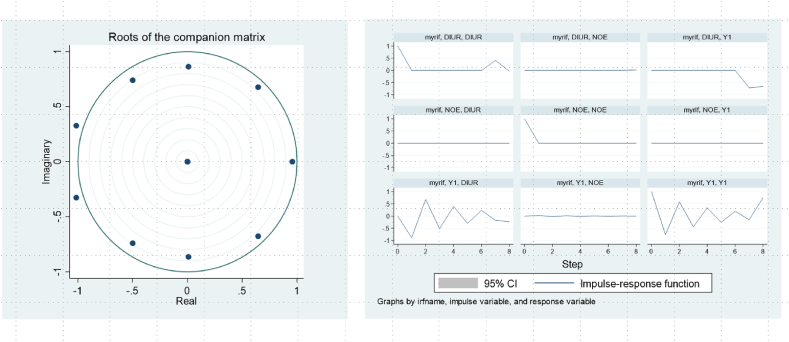
VAR model robustness test and impulse response function plot.

This investigation rigorously examines the dataset and demonstrates that altering the time frame impacts the conclusions. Therefore, the approach is modified by reducing the period under analysis. Although the adjusted linear regression data produces results that deviate somewhat from previous studies, they remain consistent within the contextual framework. Moreover, the findings exhibit an acceptable margin of error in quadratic tests conducted using alternative regression models, thereby confirming the scientific validity of the study. AA's comprehensive methodology integrates the Vector Autoregressive (VAR) model and other techniques, including impulse response functions and variance decomposition. The VAR model is specifically adept at predicting interconnected time series systems and analyzing the dynamic impact of stochastic perturbations on the set of variables. This application serves to estimate the dynamic relationships among endogenous variables collectively. Notably, this approach safeguards against 'pseudo-regressions' and guarantees the model's accuracy and the estimation results' reliability. In addition, the commonly used Augmented Dickey-Fuller (ADF) test is utilized to assess the stability of the model and the reliability of the estimation outcomes. The stationarity tests are performed on eight specific data series, encompassing aspects such as disposable income per urban resident (DIUR), urban-rural income gap (IGBR), consumer price index (CPI), commodity price index (KPI), urban unemployment rate (UUR), the old-age dependency ratio (TEDR), the juvenile dependency ratio (JPR), internet users, and the number of websites (NOE). Furthermore, supplementary tests are conducted for the regional economy (Y1) and carbon emission reduction (Y2) (see Fig. 4 for detailed information).

The test results demonstrate that the unit root hypothesis is not rejected for any of the original series, suggesting that all variables exhibit non-stationary time series behaviour. After first-order differencing, all eight series exhibit smoothness at the 1% significance level, implying they are all first-order integrated series suitable for cointegration analysis. Hence, the findings derived from the model possess scientific validity and contribute substantial scientific data and theoretical support.

### 5.3 Discussion

Staying stable and sustainable economic growth requires implementing short-term contingency policies to ensure stability, harnessing the digital dividend, leveraging endogenous demand, and fostering long-term economic development momentum. This study delves into the fundamental dynamics of the digital economy and its impact on carbon emissions by exploring crucial concepts and policies associated with this domain. The research uncovers the influence of various components within the digital economy on both these critical factors. The study's findings underscore that shifting from traditional production to the digital economy equips companies with a distinctive competitive edge, transforming the essence of products and generating new forms of value within the digital ecosystem. Furthermore, the study thoroughly examines the benefits and essential attributes of the digital economy, including information dissemination, temporal and spatial data creation, and resource allocation optimization through sharing. This comprehensive analysis contributes significantly to the broader understanding of the interplay between the digital economy and environmental sustainability, shedding light on the pathways for fostering a more environmentally conscious and digitally driven economic landscape.

Furthermore, the study delves into examining the influence of digital technology on green innovation, shedding light on how the digital economy can transcend temporal and spatial constraints, thereby facilitating the efficient transfer of technology to drive carbon reduction efforts. The research underscores the evident spillover effects of digital technology, emphasizing the critical role of innovative technology and knowledge sharing in this process. Notably, except for the urban-rural income gap (IGBR) and the urban unemployment rate (UUR), all independent variables positively impact consumption levels. The disposable income of urban residents (DIUR), consumer price index (10.13039/501100013430CPI), commodity price index (KPI), the old-age dependency ratio (TEDR), the juvenile dependency ratio (JPR), and the number of internet users (NOE) significantly contribute to regional economic development, providing partial support for our model hypothesis. The compression of spatial and temporal distances emerges as a significant characteristic of the digital economy, amplifying the scope and depth of regional economic activities through efficient information transfer. The evolution of the digital economy has shifted from a “multi-point" approach centered around pioneer regions to a “cluster" approach, with the radiation effect of the digital economy in the eastern region increasingly emphasizing the economic benefits and Western impact on regional development. This research contributes valuable insights into the intricate interplay between the digital economy and environmental sustainability, providing a nuanced understanding of the mechanisms underlying the promotion of green innovation within this context.

Moreover, all variables, except the disposable income of urban residents (DIUR) and the urban unemployment rate (UUR), exhibit a negative correlation with the level of carbon emissions. The income gap between urban and rural areas (IGBR), disposable income of urban residents (DIUR), consumer price index (CPI), commodity price index (KPI), the old-age dependency ratio (TEDR), the juvenile dependency ratio (JPR), and the number of internet users (NOE) contribute significantly to the reduction of regional carbon emissions. This study rigorously examines the impact mechanisms of the digital economy on carbon emissions reduction, both directly and indirectly. The analysis focuses on the direct effect of the digital economy itself in reducing carbon emissions, the indirect effect of the digital economy in curbing carbon emissions through upgrading the industrial structure, and the complementary effect achieved through the establishment of carbon markets, among other factors. This comprehensive investigation confirms the digital economy's substantial spillover effect and critical role in driving regional and national carbon reduction efforts. The findings underscore the multifaceted impact of the digital economy on environmental sustainability, elucidating its capacity to facilitate a transition towards greener and more sustainable economic practices.

## 6 Conclusion and policy implications

### 6.1 Research conclusions

This comprehensive study rigorously investigates the digital economy's direct and indirect impacts on regional economic development and carbon emissions, utilizing extensive data from Qinghai, a province in western China. The research systematically explores how the digital economy facilitates the transition toward environmental sustainability. It addresses the complex endogeneity issue and fills a critical research gap regarding its role in reducing carbon emissions. Additionally, the study conducts a nuanced spatial analysis of the digital economy's influence on carbon emissions, employing a multifaceted multiple linear regression approach to uncover non-linear relationships. By revealing diverse contributions and correlation characteristics of the digital economy concerning carbon emissions, the research offers valuable insights for formulating relevant policies. The proposed multi-level empirical analysis methodology significantly enhances the robustness and validity of the findings. Beyond examining the linear benefits of the digital economy on regional economic development, the study delves into intricate mechanisms through which the digital economy impacts carbon emissions, contributing to the mitigation of greenhouse gas emissions and environmental preservation. It identifies various crucial factors influencing the digital economy's impact on carbon reduction, including the pivotal role of digital economy development in pursuing dual carbon reduction objectives. These factors encompass the “direct effect" of reducing carbon emissions within the digital sector itself, the “indirect effect" of driving other sectors to adopt low-carbon practices, and the “complementary effect" achieved through the establishment of integrated carbon markets and other strategic initiatives.

Foremost among the current imperatives is the swift advancement of infrastructure development, focusing on establishing a new generation of digital infrastructure. This initiative must prioritize data elements' acceleration, marketization, and commercialization. The Western region, renowned for its abundant photovoltaic resources, has a unique opportunity to catalyze the expansion of distributed energy systems through the seamless integration of digital technology. This integration aims to achieve interoperability among various compact integrated energy supply stations. By utilizing digital technology as an intermediary and establishing the energy internet as a platform, energy silos can be mitigated, facilitating the seamless integration of digital and energy systems. Establishing an inter-provincial mechanism for exchanging and sharing digital economy resources is paramount. Such an initiative would amplify the spatial spillover effect of the digital economy, enhancing the mobility of digital elements and promoting the synchronization and coupling of data and energy, ultimately leading to a significant improvement in energy efficiency. Prioritizing the balanced and robust development of the digital economy across the western provinces of China is pivotal, given its potential to stimulate regional economic growth and foster sustainable and inclusive development in the region.

Secondly, there is an urgent need to strengthen industrial ecology and move beyond the conventional narrow perspective of regional industrial development. Aligned with China's “dual carbon" objectives, the development of digital economy infrastructure, the adoption of new energy sources, and the transformation and digitization of industries are foundational for achieving the ambitious goal of carbon neutrality. It is crucial to seamlessly integrate digital technology across sectors such as construction, coal and electricity, transportation, and chemical industries, thereby fostering the technological modernization of traditional endeavors. Coordinated efforts must be directed toward establishing extensive clusters and markets for high-end manufacturing. The emphasis should be on a novel manufacturing ecological system characterized by intelligent interconnectedness and sustainable integration. This approach will lay the groundwork for creating a robust manufacturing agglomeration pattern driven by innovation and facilitated by the synergy of digital technology and regional comparative advantages. Prioritizing the integration of digital innovation within the industrial landscape holds the potential to significantly propel the western provinces of China toward a trajectory of sustainable and technologically advanced development.

Thirdly, emphasizing innovation in institutional mechanisms is of paramount importance. A strategic focus on promoting the reform and innovation of institutional structures is imperative. It encompasses optimizing digital resource allocation, reinforcing the safeguarding of digital rights and interests, and cultivating a robust ecosystem for nurturing digital talents. Significant investments must be directed toward digital economy education and training to enhance professionals' digital literacy and application capabilities. Strengthening both the “hard guarantees" provided by institutional mechanisms and the “soft strength" derived from quality enhancement and literacy development within the digital economy is pivotal for fostering the high-quality development of manufacturing industries. As a vital instrument in the pursuit of carbon neutrality, the digital economy necessitates comprehensive collaboration, particularly in bolstering digital industrialization, driving the digitization of industries, and effecting the transformation of traditional industries through digital innovation and implementing green, low-carbon initiatives. Moreover, a systematic advancement of the manufacturing industry in Western China into a new era of the digital economy and a heightened phase of high-quality development is imperative. It demands a concerted focus on multifaceted aspects such as structural adjustments, industrial upgrading, and the cultivation of an enabling environment that encourages the seamless integration of digital technologies within the manufacturing landscape.

The research conducted for this paper has underscored the crucial need for further exploration in the digital economy domain. Our findings have revealed the pivotal role of the digital economy in restructuring global factor resources, reshaping the international economic framework, and transforming the global competitive landscape. It stands as a key driver in the contemporary wave of global competition. Beyond its industrial and technological significance, the digital economy emerges as a potent force propelling social progress and advancing human civilization. It serves as a robust platform for enhancing the efficiency and quality of traditional sectors, fostering the development of nascent industries and growth sectors, and creating pathways for employment opportunities and wealth generation. Moreover, the digital economy is a dynamic platform meeting escalating societal demands for improved living standards while simultaneously catalyzing and enhancing the quality and efficiency of government services. It represents a vehicle for fostering social stability and security, embodying a multifaceted mechanism underpinning the nuanced progression of modern societies.

### 6.2 Implications, limitations, and future research

While this paper has aimed to thoroughly investigate the intricate relationship between the digital economy, regional economic development, and carbon emissions, it is crucial to acknowledge certain limitations inherent in the research. Primarily, the data used in this study span from 2006 to 2020. The application of principal component analysis and cluster analysis to assess the data was limited to the initial phases of the COVID-19 pandemic due to the inherent time lag between data collection and dissemination. Further data support is essential to comprehensively examine the pandemic's precise impact, requiring future research to encompass a more dynamic assessment of each variable's temporal evolution.Additional variables were incorporated to enhance the model's explanatory capacity, allowing the construction of a more intricate and dynamic analytical framework. However, including these supplementary variables may introduce a certain level of complexity, thereby warranting a comprehensive examination of their implications in subsequent investigations.Expanding the temporal and spatial dimensions of the study could provide a more comprehensive understanding of the digital economy's multifaceted impact on regional economic development and carbon emissions. Complete and precise data and sophisticated analytical tools are vital for obtaining a nuanced understanding of the dynamic interactions between the digital economy, regional economies, and carbon emissions. It facilitates the formulation of robust policies and strategic interventions for sustainable development.

Exploring the ideas and limitations of studying the digital economy constitutes a multifaceted and expansive field. Diverse perspectives and methodologies adopted by various research institutions and academics underscore the need for a standardized and comprehensive understanding of the concept. The absence of a universally accepted definition and scope for the “digital economy" presents a significant challenge, leading to ambiguity and varied interpretations. Furthermore, imperfections in accounting practices related to the “digital economy," arising from the absence of common standards and norms, complicate the assessment of its impact.Inadequate and fragmented data sources about the “digital economy" hinder comprehensive analysis, highlighting the urgent need for an efficient data collection and integration mechanism. The lack of international coordination and alignment results in non-comparable accounting outcomes for the “digital economy," inhibiting the ability to draw meaningful global comparisons.In light of these constraints, our commitment remains unwavering as we persist in the pursuit of clarifying the concept of the “digital economy" through a robust definition and establishing consensus and recognition within the academic and policy communities. We aim to enhance “digital economy accounting methods" by formulating a scientific and rational index system, ensuring accuracy and reliability in the assessment process. Additionally, we prioritize the comprehensive utilization of “digital economy data sources" by developing an efficient and dependable data platform that facilitates streamlined data collection and analysis. Our ongoing efforts seek to foster a conducive environment for further research and policy development in the digital economy domain, ultimately contributing to the advancement and sustainability of this vital sector.

## Data availability statement：

The research data will not be provided separately in this paper, but the corresponding author can be contacted if needed. Data will be made available on request.

## Fund project

This research has received funding from.

1.Qinghai Minzu University: (NO:001)Outstanding Achievements of the Fifth Dr Tianqing Forum Funding for Achievements Qinghai Minzu University.
2.Qinghai Minzu University: (NO:39D2023004)Study on the practical path of promoting green, low-carbon and circular development in Qinghai Province.
3.Tianjin Research Innovation Project for Postgraduate Students: (2022BKYZ029)The linkage of technology-organization-environment condition Configuration on the construction of low carboncity in China's development performance.

## CRediT authorship contribution statement

**Tian Sun:** Writing – review & editing, Writing – original draft, Methodology, Investigation, Conceptualization. **Kaisheng Di:** Writing – review & editing, Writing – original draft, Visualization, Validation, Supervision, Methodology, Funding acquisition, Data curation, Conceptualization. **Qiumei Shi:** Investigation, Funding acquisition, Formal analysis, Data curation.

## Declaration of competing interest

The authors declare that they have no known competing financial interests or personal relationships that could have appeared to influence the work reported in this paper.
